# Case Report: Sequential eculizumab and belimumab in a paediatric patient with lupus nephritis and immune complex-mediated renal microangiopathy

**DOI:** 10.3389/fimmu.2026.1808435

**Published:** 2026-08-10

**Authors:** Ruiqi Zhu, Meng Liu, Xuechan Huang, Yuexi Zhang, Huanying He, Jiali Ding, Zhengping Huang, Shaoling Zheng, Tianwang Li

**Affiliations:** 1 Department of Rheumatology and Immunology, The Affiliated Guangdong Second Provincial General Hospital of Jinan University, Guangzhou, China; 2 The Second People’s Hospital of Panyu District, Guangzhou, China; 3 Department of Rheumatology and Immunology, Sihui People’s Hospital, Zhaoqing, China

**Keywords:** belimumab, eculizumab, lupus nephritis, renal microangiopathy, systemic lupus erythematosus

## Abstract

**Background:**

Renal microangiopathy is a severe complication of systemic lupus erythematosus, particularly when mediated by immune complex deposition. The optimal management of paediatric immune complex-mediated renal microangiopathy with rapid clinical deterioration remains unclear, and data on sequential complement and B-cell targeted therapy in this setting are scarce.

**Case summary:**

A previously healthy 14-year-old Chinese male patient presented with joint pain and generalised oedema. He was diagnosed with systemic lupus erythematosus, and the renal biopsy confirmed lupus nephritis (IV combined with V type) and immune complex-mediated renal microangiopathy (IC-RMA). The patient received a first-line intensive treatment regimen and showed an insufficient clinical response. Considering the severity and suspected complement-driven injury, we then initiated eculizumab, and his joint pain, generalised oedema, haematuria, proteinuria, and SLEDAI score were all improved. Belimumab was subsequently administered after 3 months of eculizumab therapy, and there was no recurrence for 1 year later.

**Conclusion:**

Sequential eculizumab and belimumab may be potential alternatives for selected paediatric patients with IC-RMA, although further studies are needed to confirm their efficacy and safety.

## Introduction

Systemic lupus erythematosus (SLE) is a chronic autoimmune disorder characterised by widespread systemic inflammation and multi-organ involvement, affecting approximately 3.4 million individuals worldwide (1). The kidney is the most commonly affected organ, with an estimated 40% of patients with SLE developing lupus nephritis (LN), and the estimation of mortality is ranging from 4.3% to 17% after ten years (2).

Renal microangiopathy (RMA) is a frequent and severe complication in patients with SLE and LN and is associated with a poor prognosis and increased risk of mortality. Its primary pathological manifestations include immune complex deposition, arteriosclerosis, thrombotic microangiopathy, non-inflammatory necrotising vasculopathy, and true renal vasculitis. The common clinical manifestations are hypertension, anaemia, and haematuria. Among these subtypes, immune complex-mediated RMA is characterised by prominent glomerular immunoglobulin and complement deposition, often leading to classical complement pathway activation and endothelial injury, which may explain its relative resistance to conventional immunosuppression. Multiple international studies have indicated that nearly 53.4 - 81.8% of LN patients have renal vascular lesions according to the renal biopsy (3, 4), in which approximately 30% showing more than two types of vascular pathology simultaneously. The 5-year renal survival rate for LN patients with concomitant RMA is only 30% to 80% (5). Evidence indicates that patients with RMA achieve partial remission less frequently (33.3% vs. 58.0%) and have higher treatment failure rates (44.4% vs. 15.2%) than those without microangiopathy (6).

Currently, the effectiveness of conventional immunosuppressive therapies for LN with IC-RMA is limited, highlighting the urgent need for more effective therapeutic strategies. This article presents the case of a 14-year-old boy with lupus complicated by IC-RMA who showed clinical improvement with sequential treatment with eculizumab and belimumab and details the clinical course and recovery before and after the intervention.

## Case report

The patient was previously healthy and had no history of hypertension, diabetes, or renal diseases. He reported no known drug allergies or history of tuberculosis or viral hepatitis.

In late October, 2024, a 14-year-old male patient from China presented with persistent right knee pain with no swelling that was aggravated during squatting and standing. Concurrently, he developed dark, tea-coloured, and frothy urine. Over the subsequent two weeks, progressive facial oedema developed, advancing to pitting oedema in both lower limbs and accompanied by abdominal distension, prompting hospital admission. Laboratory tests revealed positive anti-nuclear antibodies, markedly elevated anti-dsDNA antibody levels, decreased complement C3 and C4 levels, and severe proteinuria (**Table 1**). Renal ultrasonography revealed diffuse parenchymal echogenicity abnormalities and mild-to-moderate ascites. Based on these findings, SLE with LN was diagnosed, and the patient received intravenous human albumin supplementation at the very first, but this produced little clinical improvement.

**Table 1:** Laboratory data and disease activity before and after treatment.

ItemTime point	Baseline	Day after Bel	Day after 5 PEX	Day of 1 week after first dose of Ecu	Day of 1 week after fourth dose of Ecu	Day before the first dose of Bel	1 year follow-up	18 months follow-up
Haemoglobin (g/L)	103	96	75	92	96	125	122	132
White blood cells (10^9^/L)	6.4	18.6	7.4	14.7	18.1	15.8	8.9	9.3
Platelets (10^9^/L)	54	269	223	367	321	312	303	314
CD3^+^ T cells (cells/μL)	733	–	–	1938	–	2081	–	3089
CD4^+^ T cells (cells/μL)	235	–	–	555	–	660	–	1225
CD8^+^ T cells (cells/μL)	485	–	–	1386	–	1469	–	2007
CD19^+^ B cells (cells/μL)	635	–	–	691	–	732	–	344
CD16^+^CD56^+^NK cells (cells/μL)	25	–	–	60	–	158	–	222
Serum creatinine (umol/L)	149.2	170.8	141	87.4	152	94.2	76.6	85.9
Blood urea nitrogen (mmol/L)	16.4	23.4	11.4	15.3	18.5	12.7	13.1	7.3
24-h urine protein (g)	8.19	19.11	17.74	13.35	1.30	–	0.06	0.02
Albumin-to-Creatinine Ratio (mg/gcr)	5097.7	7796.6	12523.6	7892.5	1075.5	492.5	14.2	18
24h β2-microglobulin (mg)	–	12.3	12.3	–	0.2	–	0.1	0.05
ANA	1:1000	1:320	–	–	1:100	–	1:100	<1:100
Anti-dsDNA antibody (IU/ml)	897.5	<10	–	–	<10	–	<10	<10
Anti-β2GPI antibody (RU/ml)	4.57	–	–	–	8.33	–	8.19	1.02
Anticardiolipin antibody (RU/ml)	8.69	–	–	–	3.64	–	3.25	12.31
Schistocytes (%)	4.9	–	–	1.3	–	–	–	0.5
Direct Coombs test	Negative	–	–	–	–	–	–	–
ADAMTS13 activity (%)	49.24%	–	–	–	–	–	–	–
ADAMTS13 inhibitor	Negative	–	–	–	–	–	–	–
Lupus anticoagulant screen ratio (dRVVT)	1.7	–	–	–	–	–	–	–
Lupus anticoagulant confirm ratio (dRVVT)	1.6	–	–	–	–	–	–	–
Normalised Lupus anticoagulant ratio	1.06	–	–	–	–	–	–	–
C3 (g/L)	0.15	0.46	0.56	0.73	0.93	1.05	0.68	1.03
C4 (g/L)	0.03	0.11	0.12	0.15	0.22	0.25	0.17	0.31
Lactate Dehydrogenase (U/L)	315	272	–	–	271	222	143	150
Erythrocyte sedimentation rate (mm/h)	–	19.6	–	–	22.0	–	5.8	5.5
C-reactive protein (mg/L)	–	1.7	–	–	0.7	–	0.5	0.3
Albumin (g/L)	24.6	13.6	20.5	23.8	21.8	32.0	31.8	40
SLEDAI	21	18	18	12	4	0	0	0

C3, complement 3; C4, complement 4; PEX, plasma exchange; Ecu, eculizumab; Bel, belimumab; SLEDAI Systemic Lupus Erythematosus Disease Activity Index.

A comprehensive diagnostic workup was performed to clarify the aetiology of renal microangiopathy. Antiphospholipid antibody testing (lupus anticoagulant, anticardiolipin, and anti-β2GPI antibodies) was negative, ruling out antiphospholipid syndrome. ADAMTS13 activity was within the normal range (49.24%), and the ADAMTS13 inhibitor was negative, excluding immune-mediated thrombotic thrombocytopenic purpura. Peripheral blood smear revealed that schistocytes accounted for 4.9% of all erythrocytes, confirming intravascular haemolysis (**Table 1**). Serial blood pressure measurements during the acute phase showed systolic readings ranging from 111 to 149 mmHg and diastolic readings from 71 to 108 mmHg, with a peak of 149/108 mmHg recorded on December 23, 2024; importantly, diastolic pressure never exceeded 110 mmHg, a threshold well below the typical range for malignant hypertension. The blood pressure normalised with diuresis and disease control, further arguing against malignant hypertension as the primary aetiology. No recent infectious prodrome was reported, blood cultures remained negative, and the patient had no history of exposure to TMA-inducing drugs such as quinine, calcineurin inhibitors at supratherapeutic doses, or anti-VEGF agents. Although infection- or drug-associated TMA cannot be entirely ruled out, the clinical picture and laboratory findings did not support these possibilities.

**Figure 1:**
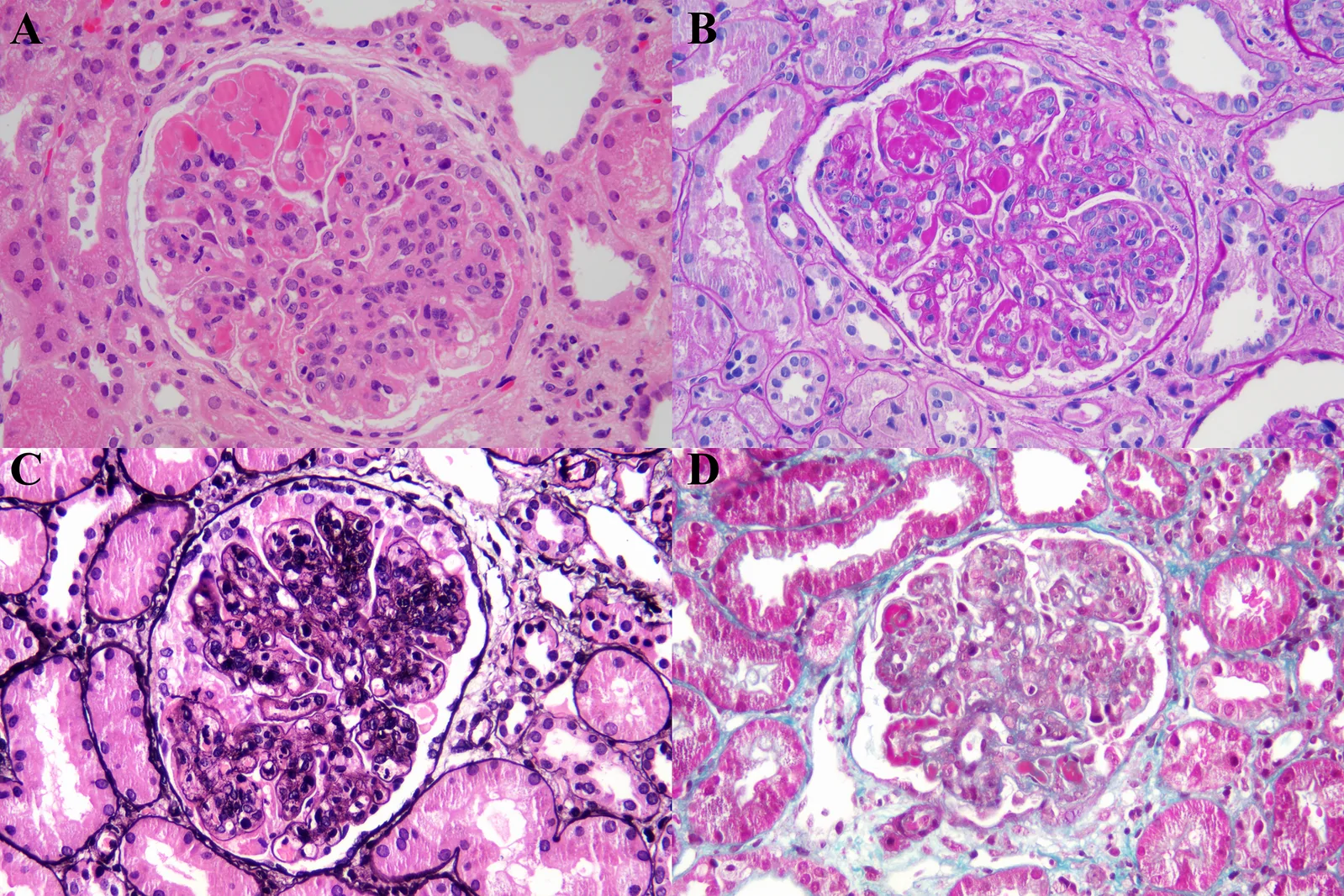
Histopathological features of renal biopsy tissue. **(A)** Glomerular capillary loops show lobular accentuation with neutrophil infiltration, karyorrhexis, and intracapillary fibrin thrombi (hematoxylin-eosin, ×400). **(B)** Diffuse mesangial and endothelial cell proliferation with focal capillary lumen compression, occlusion, and small cellular fibrous crescent formation (periodic acid-Schiff, ×400). **(C)** Glomerular basement membranes exhibit segmental thickening with a rigid appearance, mesangial interposition, and double-contour formation (periodic acid-silver methenamine, ×400). **(D)** Mesangial, subendothelial, and subepithelial eosinophilic deposits with “wire-loop” lesions (Masson trichrome, ×400).

**Figure 2:**
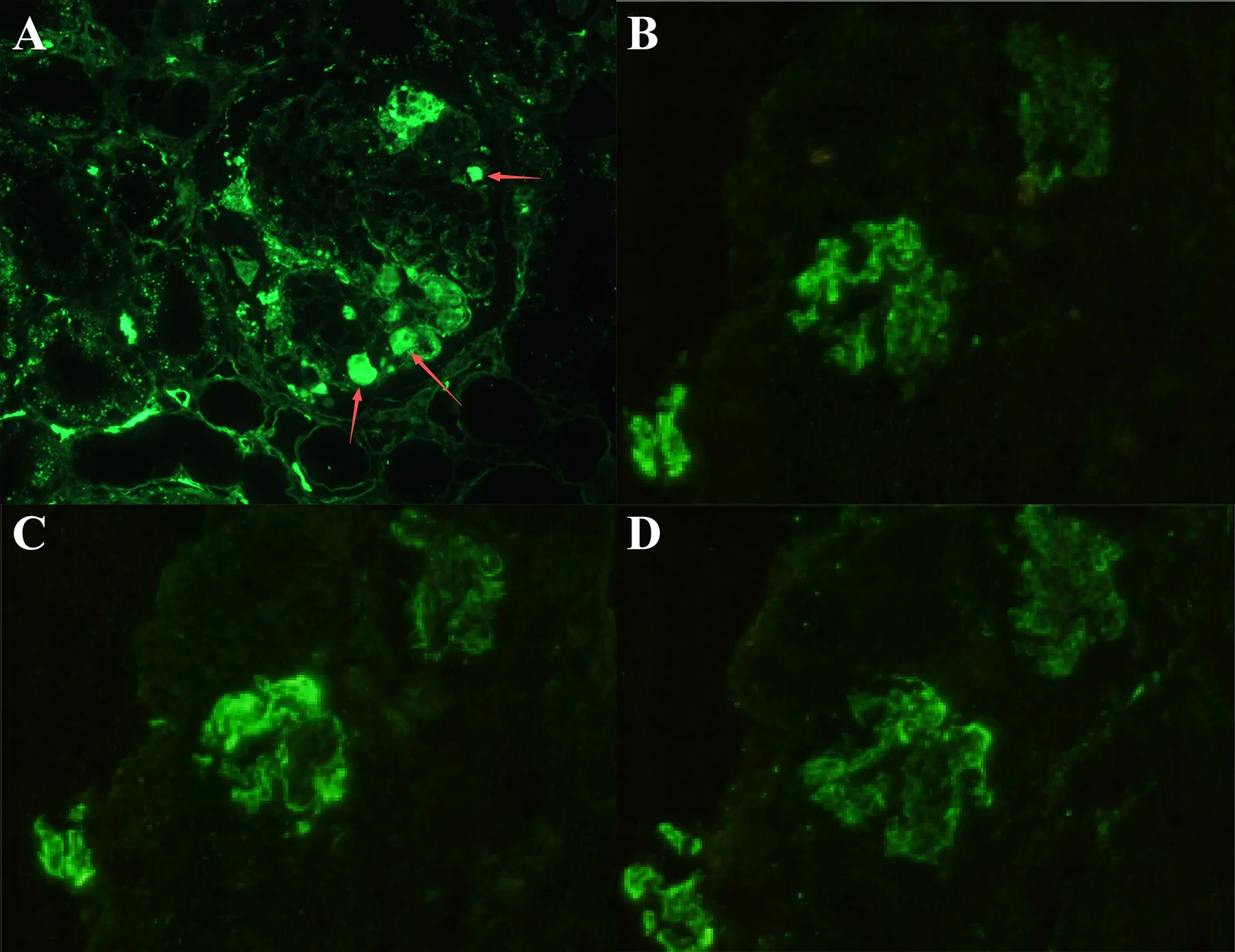
Immunofluorescence staining of renal biopsy tissue. **(A)** IgG deposition; **(B)** C3 deposition; **(C)** IgA deposition; **(D)** C1q deposition. Glomerular capillary loops show diffuse granular deposits of immunoglobulins and complement components, with mesangial and peripheral capillary wall positivity. Original magnification: ×400. Scale bar:50μm.

A renal biopsy demonstrated lobular reorganisation of glomerular capillary loops, diffuse mesangial and endothelial hypercellularity, partial luminal compression and occlusion of capillaries, basement membrane thickening, occasional spike formations, segmental mesangial matrix expansion, double contour formation, deposition of basophilic material in the mesangium, eosinophilic bodies, nuclear debris, and microthrombi within some glomerular capillaries (**Figure 1**), and positive immunofluorescence for IgG, IgA, C3, and C1q (**Figure 2**). This immunofluorescence pattern is a key distinguishing feature. In primary atypical haemolytic uraemic syndrome, immunofluorescence is typically negative or shows only C3 deposition without significant immunoglobulin staining. The prominent deposition of C1q, together with IgG, IgA, and C3, is characteristic of immune complex-mediated injury rather than primary atypical haemolytic uraemic syndrome. These histopathological features were consistent with those of class IV + V lupus nephritis (activity index, 12; chronicity index, 0), complicated by IC-RMA. The patient was then administered intravenous methylprednisolone (80 mg daily for 3 days), hydroxychloroquine, diuretics, and supportive care. The oedema partially improved, and the patient was discharged with the continuation of oral glucocorticoid therapy.

On 4 December, 2024, the patient was readmitted with generalised oedema, haematuria, and persistent urinary foaming. Therapy was intensified with intravenous methylprednisolone, hydroxychloroquine was continued, oral mycophenolate mofetil and cyclosporine A were newly added, and belimumab (480 mg intravenous infusion on 11 December) was initiated. Although the arthralgia resolved, there was no significant improvement in systemic oedema or laboratory markers (**Table 1**).

Subsequently, serum creatinine and urinary protein excretion levels increased continually (**Table 1**). Despite the sequential administration of pulse therapy, comprising intravenous immunoglobulin 20 g daily and methylprednisolone 500 mg daily from 19 December to 21 December, 2024, five sessions of plasma exchange (PEX) and continuous renal replacement therapy (CRRT) were initiated on 24 December, 2024. Although the serum creatinine levels decreased, generalised oedema, haematuria, and nephrotic-range proteinuria persisted.

Given the insufficient clinical response to these therapies and the patient’s progressive deterioration with worsening oedema, increasing ascites, new-onset mild-to-moderate pleural effusion, rising proteinuria, and persistent haematuria, targeted complement inhibition with eculizumab was initiated on 4 January, 2025, at a dose of 600 mg weekly via intravenous infusion. The patient had received MenACWY and MenB vaccines approximately one year before, as required prior to complement inhibitor therapy. To minimise the risk of infection during complement blockade, we implemented a rigorous surveillance protocol throughout the treatment course, which included daily clinical assessment for fever or signs of infection, regular chest computed tomography, and frequent laboratory monitoring, including complete blood count, C-reactive protein level, and erythrocyte sedimentation rate. Additionally, given the prolonged exposure to high-dose glucocorticoids and multiple immunosuppressants, including mycophenolate mofetil and cyclosporine A, prophylaxis for p. jirovecii pneumonia was initiated with trimethoprim-sulfamethoxazole at a dose of 5 mg/kg/day, based on the trimethoprim component, and continued throughout the intensive immunosuppressive period. This intervention led to a marked improvement in clinical symptoms, including a significant reduction in oedema of the limbs and trunk, whilst facial swelling remained the same. On 13 January, 2025, prednisone was tapered from 80 mg to 40 mg daily, cyclosporine A was increased to 150 mg daily, hydroxychloroquine was maintained at 0.2 g twice daily, and mycophenolate mofetil was escalated to 75 mg twice daily.

After one month of eculizumab treatment, his facial oedema and laboratory abnormalities gradually improved (**Table 1**). The treatment protocol was further adjusted as follows: Eculizumab dosing was modified to 900 mg every two weeks for four doses; prednisone was reduced from 40 mg to 30 mg daily; cyclosporine A was decreased from 150 mg to 125 mg daily; mycophenolate mofetil was reduced from 75 mg twice daily to 50 mg twice daily; and the hydroxychloroquine dosage remained unchanged.

**Figure 3:**
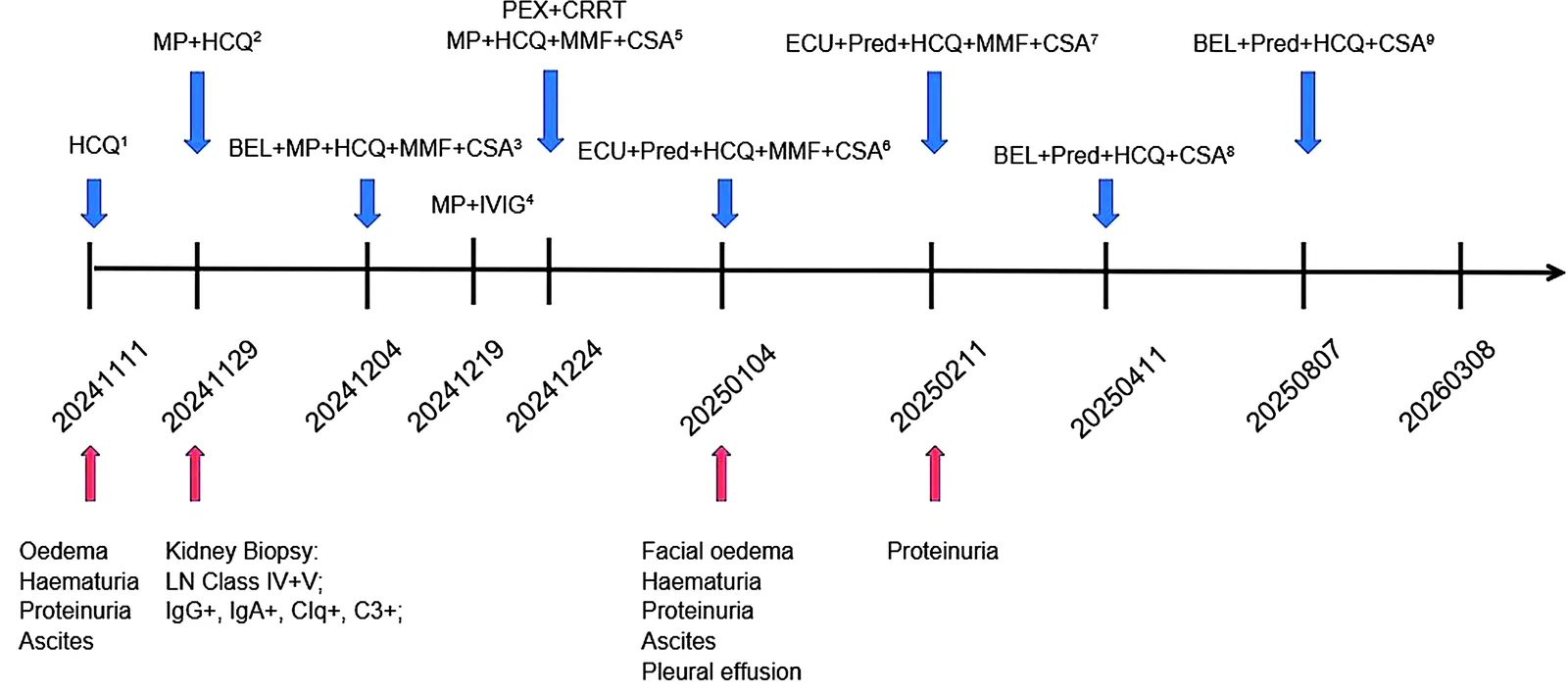
Treatment timeline and clinical course of a paediatric SLE patient with refractory renal microangiopathy. BEL, belimumab; MP, methylprednisolone; HCQ, hydroxychloroquine; PEX, plasma exchange; CRRT, continuous renal replacement therapy; ECU, eculizumab; Pred, prednisone; CSA, cyclosporine A; MMF, mycophenolate mofetil; LN, lupus nephritis. (^1^HCQ 0.2g daily, ^2^MP 80mg daily+HCQ 0.2g daily, ^3^BEL 480mg every two weeks+MP 40mg daily+HCQ 0.2g daily+MMF 150mg daily+CSA 150mg daily, ^4^MP 500mg daily+IVIG 20g daily for 3 days, ^5^MP 80mg daily+HCQ 0.2g daily+MMF 125mg daily+CSA 125mg daily, ^6^ECU 600mg weekly+Pred 80mg daily+HCQ 0.2g daily+MMF 125mg daily+CSA 125mg daily, ^7^ECU 900mg every two weeks+Pred 30mg daily+HCQ 0.2g daily+MMF 100mg daily+CSA 125mg daily, ^8^BEL 480mg every two weeks for the first three doses then BEL 480mg monthly+Pred 10mg daily+HCQ 0.2g daily+CSA 100mg daily, ^9^BEL 480mg monthly+Pred 5mg daily+CSA 75mg daily+HCQ 0.2g daily).

After three months of eculizumab therapy, the patient reported complete resolution of most symptoms. Follow-up testing revealed normalisation of the haemoglobin, serum creatinine, complement C3 and C4 levels, erythrocyte sedimentation rate, and lactate dehydrogenase (**Table 1**). Eculizumab was discontinued, and several factors influenced our decision to choose belimumab as maintenance therapy. The patient’s peripheral B-lymphocyte count remained at a high level despite clinical improvement, with CD19-positive B cells at 732 cells/μL, suggesting ongoing B-cell activation that could be therapeutically targeted. In the third month of eculizumab therapy, the patient developed a mild productive cough without fever. Chest computed tomography revealed no infiltrates, and sputum cultures were negative. The symptoms resolved spontaneously without antibiotic intervention; however, this event raised clinical concerns regarding the risk of infection under sustained complement blockade and contributed to the decision to transition to belimumab. Belimumab was therefore initiated at standard dose (480 mg intravenous infusion according to the protocol), whilst oral medications were adjusted to prednisone 10 mg daily, cyclosporine A 100 mg daily, and hydroxychloroquine 0.2 g daily. Following the tapering of the immunosuppressive regimen, the patient remained clinically stable with no recurrence of the hallmark SLE manifestations. Thereafter, a gradual tapering of immunosuppressive agents was implemented, with close monitoring over a 12-month follow-up period. The detailed therapeutic timeline is summarised in **Figure 3**. No severe adverse events were observed during this period. The patient did not experience disease recurrence throughout follow-up. The patient reported a significant improvement in his overall well-being, stating that he felt “completely back to normal” and no longer restricted by fatigue or joint discomfort. At the time of the most recent assessment on March 8, 2026 (12 months after the last eculizumab dose), the patient remained in stable remission with an SLEDAI score of 0.

## Discussion

This case report describes the case of a 14-year-old male adolescent with SLE-associated LN coexisting with IC-RMA. RMA is a recognised vascular complication of LN, characterised by diverse histopathological features that may involve immune complex deposition and thrombotic microangiopathy, amongst other vascular changes, requiring careful differentiation based on distinct pathological criteria (7). In this case, the histological findings were consistent with immune complex-driven microvascular injury, marked by extensive glomerular deposition of immunoglobulins and complement components (IgG, C3, C1q, and IgA). Differential diagnoses include complement-mediated atypical haemolytic uraemic syndrome, antiphospholipid syndrome, thrombotic thrombocytopenic purpura, malignant hypertension, infection/drug-associated TMA, and secondary TMA in SLE. As detailed above, our workup excluded APS, TTP, and malignant hypertension based on serology and blood pressure. The absence of infection and culprit drugs mitigated the alternative aetiologies. Prominent immune complex deposition with C1q positivity distinguishes this condition from primary atypical haemolytic uraemic syndrome, in which immunofluorescence is usually negative or shows only C3 deposition without significant immunoglobulin staining. Although the patient’s presentation fits the broad category of secondary thrombotic microangiopathy in SLE, we specifically characterised it as IC-RMA. This distinction is critical because IC-RMA, unlike other secondary TMA forms, is driven by glomerular immune complex deposition and classical complement activation, rather than by non-specific endothelial stress, hypertension, or systemic inflammation. This mechanistic specificity guides the differential diagnosis and supports the use of eculizumab for targeted complement blockade.

IC-RMA is strongly associated with complement activation (8). The deposition of immune complexes can activate the classical complement pathway, resulting in the formation of the terminal complement complex C5b-9, which induces direct endothelial cell damage. This mechanism may partially account for the inadequate response to conventional immunosuppressive regimens observed in some LN patients (9). Evidence supporting complement system activation in this case comprised three key observations: (i) immunofluorescence revealed glomerular deposition of C1q and C3 (**Figure 2**); (ii) diminished serum C3 and C4 concentrations (C3 at 0.15 g/L, C4 at 0.03 g/L) indicated complement consumption; (iii) complement levels rapidly normalised within one week post-eculizumab administration (C3 rose to 0.93 g/L, C4 to 0.22 g/L), thus implicating microangiopathic haemolytic anaemia as the probable pathogenic mechanism consistent with the diagnosis of IC-RMA. It is well recognised that C1q deposition on renal biopsy is a marker of classical complement pathway activation (10). Moreover, Lin et al. reported that patients with LN who present with such vascular abnormalities may have worse renal outcomes (11). Eculizumab attenuates terminal complement–mediated inflammation and cellular injury (12). While primarily approved for classic thrombotic microangiopathies such as atypical haemolytic uraemic syndrome (13), its use has been extended to SLE and LN–associated microangiopathy, particularly in cases driven by immune complex deposition and dysregulated complement activation. A systematic review by Wright et al. summarised that amongst 30 SLE patients with TMA treated with eculizumab, 93% achieved favourable outcomes, including symptom resolution and renal function recovery (14). The 2024 Kidney Disease: Improving Global Outcomes (KDIGO) Clinical Practice Guideline for Glomerular Diseases discusses eculizumab in the context of LN-associated complement-mediated microangiopathy, noting that terminal complement blockade can be considered as a rescue therapy based on a mechanistic rationale (15). In this patient, eculizumab therapy led to rapid normalisation of complement levels, reduction in proteinuria (from 17.74 g/day to 13.35 g/day within one week), and restoration of renal function (16). Notably, severe disease relapse can occur in paediatric patients following eculizumab discontinuation, as supported by studies on both LN and atypical haemolytic uremic syndrome (16, 17). Moreover, the prolonged use of eculizumab carries a significantly increased risk of serious infections and poses an economic burden. Consequently, once acute complement-mediated damage is controlled, the therapeutic focus should shift towards inducing and maintaining durable immunological remission.

Belimumab, a human monoclonal antibody that neutralises B lymphocyte stimulator, suppresses the survival, activation, and differentiation of autoreactive B cells, thereby reducing pathogenic autoantibody production (18). The Phase III Belimumab International Study in Lupus Nephritis (BLISS-LN) demonstrated that adding belimumab to standard immunosuppressive therapy significantly improved complete and partial renal response rates in active LN (19). A *post-hoc* analysis of the BLISS-LN trial further demonstrated that belimumab significantly improved eGFR recovery in patients with impaired renal function at baseline, supporting its role in long-term renal preservation (20). In this patient, several factors influenced the decision to choose belimumab as maintenance therapy: the peripheral B-lymphocyte count remained at a high level despite clinical improvement; the patient developed cough and other suspected respiratory tract infections during eculizumab therapy, raising concerns about infection risk associated with continued complement blockade; and the family faced significant financial constraints. Transitioning to belimumab-based maintenance therapy allowed progressive tapering of oral prednisone from 40 mg/day to 5 mg/day, consistent with the stepwise reduction schedule recommended by the 2023 American College of Rheumatology/European Alliance of Associations for Rheumatology LN management guidelines (21). Renal stability was maintained, and no clinical or serological flares were observed throughout the follow-up period.

The present case has several strengths. First, data on this sequential therapeutic protocol in Asian children are limited; our report provides detailed clinical information from this under-represented population. Second, the optimal duration of eculizumab therapy for paediatric IC-RMA remains undefined. Our experience with a three-month regimen, subsequently bridged to belimumab maintenance, offers a potential reference for clinicians facing similar therapeutic choices. Third, the patient presented with a life-threatening course that required dialysis at disease onset yet achieved a favourable outcome, suggesting that the sequential strategy may be viable even in severe cases. Fourth, we provide a detailed 12-month longitudinal dataset documenting renal function recovery, corticosteroid tapering, complement dynamics, and safety monitoring. While preliminary, these observations may assist clinicians in managing similarly complex paediatric cases.

This study has several limitations. Firstly, this was a single-patient, single-centre study. The safety and efficacy of this sequential treatment approach require confirmation through prospective studies or larger cohorts. Secondly, because multiple concomitant therapies were administered, the causality cannot be definitively attributed to any single agent. Additionally, more specific complement biomarkers (e.g., C4d or C5b-9 deposition on biopsy, or urinary complement activation products) were not available, which might have provided additional evidence (22). Their assessment in future studies may help guide personalised therapeutic strategies. Finally, given the exploratory nature of this single case, all findings should be interpreted with caution and require validation in larger cohorts.

## Conclusion

In paediatric patients with active LN and IC-RMA and rapid clinical progression despite conventional therapy, a short course of eculizumab may serve as a potential salvage option. The subsequent transition to belimumab for long-term maintenance, combined with an aggressive glucocorticoid-reduction strategy, may lead to sustained remission and reduce the risk of treatment-related complications. This sequential approach warrants further investigation in larger prospective cohorts and should be limited to carefully selected patients.

## Data Availability

The original contributions presented in the study are included in the article/supplementary material. Further inquiries can be directed to the corresponding authors.

## References

[1] Tian JR Zhang DY Yao X Huang YQ Liu QJ . Global epidemiology of systemic lupus erythematosus: a comprehensive systematic analysis and modelling study. Ann Rheum Dis. (2023) 82:351–6. doi: 10.1136/ard-2022-223035 36241363 PMC9933169

[2] Hocaoğlu M Valenzuela-Almada MO Dabit JY Osei-Onomah SA Chevet B Giblon RE . Incidence, prevalence, and mortality of lupus nephritis: a population-based study over four decades using the lupus midwest network. Arthritis Rheumatol. (2023) 75:567–73. doi: 10.1002/art.42375 36227575 PMC10065880

[3] Wu LH Yu F Tan Y Qu Z Chen MH Wang SX . Inclusion of renal vascular lesions in the 2003 ISN/RPS system for classifying lupus nephritis improves renal outcome predictions. Kidney Int. (2013) 83:715–23. doi: 10.1038/ki.2012.409 23302713

[4] Barber C Herzenberg A Aghdassi E Su J Lou W Qian G . Evaluation of clinical outcomes and renal vascular pathology among patients with lupus. Clin J Am Soc Nephrol. (2012) 7:757–64. doi: 10.2215/CJN.02890311 22442181 PMC3338275

[5] Ding Y Tan Y Qu Z Yu F . Renal microvascular lesions in lupus nephritis. Ren Fail. (2019) 42:19–29. doi: 10.1080/0886022X.2019.1702057 31858861 PMC6968586

[6] Sun F Wang X Wu W Wang K Chen Z Li T . TMA secondary to SLE: rituximab improves overall but not renal survival. Clin Rheumatol. (2018) 37:213–8. doi: 10.1007/s10067-017-3793-4 28856466

[7] Bomback AS Appel GB . Updates on the treatment of lupus nephritis. J Am Soc Nephrol. (2010) 21:2028–35. doi: 10.1681/ASN.2010050472 21051743

[8] Lech M Anders HJ . The pathogenesis of lupus nephritis. J Am Soc Nephrol. (2013) 24:1357–66. doi: 10.1681/ASN.2013010026 23929771 PMC3752952

[9] Noris M Remuzzi G . Glomerular diseases dependent on complement activation, including atypical hemolytic uremic syndrome, membranoproliferative glomerulonephritis, and C3 glomerulopathy. Am J Kidney Dis. (2015) 66:359–75. doi: 10.1053/j.ajkd.2015.03.040 26032627 PMC4528072

[10] Koopman JJE Rennke HG McMahon GM Waikar SS Costenbader KH . Associations between renal deposits of complement factors, disease activity and loss of renal function in lupus nephritis. J Clin Pathol. (2021) 75:789–90. doi: 10.1136/jclinpath-2021-207788 34969783 PMC10015506

[11] Lin KY Chan EY Mak YF To MC Wong SW Lai FF . Renal vascular lesions in childhood-onset lupus nephritis. Pediatr Nephrol. (2025) 40:131–41. doi: 10.1007/s00467-024-06498-z 39249126 PMC11584461

[12] Legendre CM Licht C Muus P Greenbaum LA Babu S Bedrosian C . Terminal complement inhibitor eculizumab in atypical hemolytic-uremic syndrome. N Engl J Med. (2013) 368:2169–81. doi: 10.1056/NEJMoa1208981 23738544

[13] Scully M Cataland SR Peyvandi F Coppo P Knöbl P Kremer Hovinga JA . Caplacizumab treatment for acquired thrombotic thrombocytopenic purpura. N Engl J Med. (2019) 380:335–46. doi: 10.1056/NEJMoa1806311 30625070

[14] Wright RD Bannerman F Beresford MW Oni L . A systematic review of the role of eculizumab in systemic lupus erythematosus-associated thrombotic microangiopathy. BMC Nephrol. (2020) 21:245. doi: 10.1186/s12882-020-01888-5 32605540 PMC7329551

[15] Kidney Disease: Improving Global Outcomes (KDIGO) Glomerular Diseases Work Group . KDIGO 2024 clinical practice guideline for the management of glomerular diseases. Kidney Int. (2024) 105:S1–S286. doi: 10.1016/j.kint.2024.03.016 38182286

[16] Coppo R Peruzzi L Amore A Martino S Vergano L Lastauka I . Dramatic effects of eculizumab in a child with diffuse proliferative lupus nephritis resistant to conventional therapy. Pediatr Nephrol. (2015) 30:167–72. doi: 10.1007/s00467-014-2944-y 25173358

[17] AlZabali S AlBatati S Rahim K Faqeehi H Osman A Bamhraz A . A multicenter study evaluating the discontinuation of eculizumab therapy in children with atypical hemolytic uremic syndrome. Children (Basel). (2022) 9:1734. doi: 10.3390/children9111734 36421183 PMC9688604

[18] Stohl W Hiepe F Latinis KM Thomas M Scheinberg MA Clarke A . Belimumab reduces autoantibodies, normalizes low complement levels, and reduces select B cell populations in patients with systemic lupus erythematosus. Arthritis Rheum. (2012) 64:2328–37. doi: 10.1002/art.34400 22275291 PMC3350827

[19] Furie R Rovin BH Houssiau F Malvar A Teng YKO Contreras G . Two-year, randomized, controlled trial of belimumab in lupus nephritis. N Engl J Med. (2020) 383:1117–28. doi: 10.1056/NEJMoa2001180 32937045

[20] Calatroni M Limoncella G Chiara E Emmi G Moroni G Lucenteforte E . Effect of belimumab on patients with lupus nephritis and impaired kidney function: post hoc subgroup analysis of the phase III BLISS-LN trial. Lupus Sci Med. (2026) 13:e001834. doi: 10.1136/lupus-2025-001834 41986079 PMC13084980

[21] Fanouriakis A Kostopoulou M Andersen J Aringer M Arnaud L Bae SC . EULAR recommendations for the management of systemic lupus erythematosus: 2023 update. Ann Rheum Dis. (2024) 83:15–29. doi: 10.1136/ard-2023-224762 37827694

[22] Kesarwani V Bukhari MH Kahlenberg JM Wang S . Urinary complement biomarkers in immune-mediated kidney diseases. Front Immunol. (2024) 15:1357869. doi: 10.3389/fimmu.2024.1357869 38895123 PMC11184941

